# Domestication of Pea (*Pisum sativum* L.): The Case of the Abyssinian Pea

**DOI:** 10.3389/fpls.2018.00515

**Published:** 2018-04-18

**Authors:** Norman F. Weeden

**Affiliations:** Department of Plant Sciences and Plant Pathology, Montana State University, Bozeman, MT, United States

**Keywords:** *Pisum sativum*, domestication, gene trees, genetic diversity, *Pisum fulvum*

## Abstract

Phylogenetic relationships of the Abyssinian pea (*Pisum sativum* ssp. *abyssinicum*) to other subspecies and species in the genus were investigated to test between different hypotheses regarding its origin and domestication. An extensive sample of the *Pisum sativum* ssp. *sativum* germplasm was investigated, including groups a-1, a-2, b, c, and d as identified by Kwon et al. (2012). A broad sample of *P. fulvum* but relatively few *P. s.* ssp. *elatius* accessions were analyzed. Partial sequences of 18 genes were compared and these results combined with comparisons of additional genes done by others and available in the literature. In total, 54 genes or gene fragment sequences were involved in the study. The observed affinities between alleles in *P.* ssp. *sativum*, *P. s.* ssp. *abyssinicum*, *P. s*. ssp. *elatius*, and *P. fulvum* clearly demonstrated a close relationship among the three *P. sativum* subspecies and rejected the hypothesis that the Abyssinian pea was formed by hybridization between one of the *P. sativum* subspecies and *P. fulvum*. If hybridization were involved in the generation of the Abyssinian pea, it must have been between *P. s.* ssp. *sativum* and *P. s.* ssp. *elatius*, although the Abyssinian pea possesses a considerable number of highly unique alleles, implying that the actual *P. s.* ssp. *elatius* germplasm involved in such a hybridization has yet to be tested or that the hybridization occurred much longer ago than the postulated 4000 years bp. Analysis of the *P. s.* ssp. *abyssinicum* alleles in genomic regions thought to contain genes critical for domestication indicated that the indehiscent pod trait was independently developed in the Abyssinian pea, whereas the loss of seed dormancy was either derived from *P. s*. ssp. *sativum* or at least partially developed before the *P. s.* ssp. *abyssinicum* lineage diverged from that leading to *P. s.* ssp. *sativum*.

## Introduction

The Abyssinian pea [*Pisum sativum* ssp. *abyssinicum* A. Br.) (alternatively: *Lathyrus schaeferi* Kosterin) (Kosterin, 2017) is a well-defined taxon of uncertain derivation. It is cultivated in Ethiopia and Yemen along with the typical domesticated pea (*Pisum sativum* L.) and appears to lack a wild form (see discussion in Kosterin, 2017). Yet it crosses only with difficulty to the cultivated pea, with wild forms of *P. sativum* (*P. s.* ssp. *elatius* (Bieb.) Schmalh.), or with the other species in the genus (*P. fulvum* Sibeth. & Sm.). The reduced fertility in these crosses appears to be due to both prezygotic and postzygotic barriers (Kosterin, 2017). In flower color and general habit the Abyssinian pea resembles the domesticated pea, although it possesses several traits rarely found in the domesticated pea, such as strongly serrate leaflets and a glossy seed coat. Relevant to the domestication of pea, it shares with *P. s.* ssp. *sativum* an indehiscent pod and absence of a seed dormancy mechanism (in *Pisum* seed dormancy is primarily controlled by a thick, water-impermeable seed coat). These two traits do not segregate in populations derived from crosses between *P. s.* ssp. *abyssinicum* and *P. s.* ssp. *sativum*), all progeny possessing indehiscent pods and producing seeds that usually germinate immediately after planting (Weeden, 2007, unpublished). Hence, the same loci must have been mutated in each lineage. Finally, genetic diversity within *P. s.* ssp. *abyssinicum* is extremely limited, (Weeden and Wolko, 2001; Vershinin et al., 2003), suggesting that the taxon has experienced a recent, severe bottleneck or is a relatively young taxon.

The combination of low genetic diversity, reproductive semi-isolation from close relatives, and numerous unique traits or markers in the Abyssinian pea has led to various alternative hypotheses about its origin. Govorov (1937) suggested that it may have been formed by hybridization between *P. sativum* and *P. fulvum*, producing plants that displayed partial sterility with both parental species. All three taxa have the same chromosome number (2n = 14) (Errico et al., 1991) but differ in karyotype or plastid/cytoplasmic compatibility group (Saccardo, 1971; Bogdanova et al., 2015). Studies with various DNA markers have identified a considerable number of markers present in *P. s.* ssp. *abyssinicum* that are much more closely related to *P. fulvum* than to *P. s*. ssp. *sativum* (Vershinin et al., 2003), supporting the hybrid hypothesis. Due to the near absence of genetic diversity in the Abyssinian pea, the hybridization event has been postulated to have occurred about 4000 years bp (Kosterin, 2017), and the traits critical for initial domestication must have been obtained from the *P. sativum* parent.

In contrast, the similarity of *P. s.* ssp. *abyssinicum* to *P. s.* ssp. *sativum* in flower color, seed size and several other morphological features, as well as the ability to make reasonably fertile crosses between the two, led many students of the genus to designate the Abyssinian pea as a subspecies of the domesticated taxa (Berger, 1928). The marked divergence of this taxon and its limited genetic diversity being attributed to its isolation and cultivation in the Ethiopian highlands. In this latter hypothesis, isolation and intense selection permits the divergence time between the two subspecies to be moved back considerably. In *Pisum*, the two traits critical for domestication (indehiscent pods and lack of seed dormancy) are influenced by several loci (Weeden et al., 2002), although one locus, *Dpo*, plays a major role in pod dehiscence (Blixt, 1972). The finding that the same loci were modified in both subspecies led the current author to suggest that the two taxa may have diverged after these mutations had occurred, probably less than 10,000 years bp (Weeden, 2007).

In the present study I attempted to distinguish between the two hypotheses described above by comparing the DNA sequences of portions of 18 genes in accessions of *P. fulvum*, and the three subspecies of *P. sativum*. This approach is very similar to that used by Jing et al. (2007), although their study focused on the genetic diversity within the genus rather than testing between the alternate hypotheses above. Hence, I was able to use their data on an additional 34 sequences to greatly expand the coverage of the genome as well as the number of accessions of *P. s. ssp. elatius* included. In addition, the data from two studies on histone gene diversity (Zaytseva et al., 2012, 2015) were likewise included in my investigation. The 54 genes are distributed across all seven chromosomes of pea, allowing any significant contribution from *P. fulvum* to the Abyssinian pea to be recognizable as a high similarity between the gene sequences in that region and those in *P. fulvum*. In contrast, if the Abyssinian pea represents a divergence from *P. s.* ssp. *sativum* after initial domestication changes, virtually all Abyssinian alleles should nest within the diversity of *P. s*. ssp. *sativum*.

## Materials and Methods

### Plant Material

Ten accessions of *P. s.* ssp. *sativum* were obtained from the USDA collection at Pullman, WA, United States (Supplementary Table S1). These accessions represented samples from each of the four major clades (a, b, c, and d) from Kwon et al. (2012). In addition, 66 modern pea varieties and two *P. s.* ssp. *elatius* accessions, JI 1794 and JI 261 were included (Supplementary Table S1). JI 1794 is a representative of the ‘northern humile’ group that is considered a likely candidate for the wild ancestor of domesticated pea. Crosses between it and domesticated lines are highly fertile. JI 261 produces partly sterile hybrids in crosses with domesticated lines, but also can be crossed with *P. fulvum*, again producing a hybrid with reduced fertility (Weeden, personal observation). Nine accessions of *P. fulvum*, representing a wide sample of the genetic diversity in this species, were generously provided by Dr. S. Abbo, The Hebrew University, Rehovot, Israel. Passport data for these can be obtained in Naim-Feil et al. (2017). Two additional samples of *P. fulvum* from Israel (VIR 6070 and VIR 6071) were kindly supplied by Dr. F. Gorel, then at the Institute of Cytology & Genetics, Novosibirsk, Russia. All *P. fulvum* samples were subjected to sequencing of four genes: *Pur* (PURPLE-PODDED), *R*_b_ (*ADP-glucose phosphorylase*), *GlyOH* (glucan endo-1,3-beta-glucosidase), and *Gpic* (cytosolic glucose phosphate isomerase). For the remaining genes, only three *P. fulvum* sequences were obtained, one from those provided by S. Abbo (usually *P. fulvum*-19) and the two VIR accessions.

### Genes Analyzed

The genes examined, the primers used and the size of the fragment sequenced are presented in Supplementary Table S2. In all cases the primers were designed to match conserved portions of the coding sequence of the gene, but the amplified segment included at least one intron.

### PCR Conditions

All PCR reactions (20 μl) contained the following: 4 μl Promega 5X PCR buffer, 2.5 mM MgCl_2_, 0.3 μM of each dNTP, 0.6 units Promega Taq polymerase, and approximately 25 ng pea genomic DNA. In all cases a touchdown procedure was employed, with annealing temperature starting at 63°C and dropping in 1° steps to the final annealing temperature listed on Supplementary Table S2.

### DNA Sequencing

PCR products were purified using QIAquick PCR Purification Kit (QIAGen), and DNA concentrations determined after purification using a Nanodrop 2000 (Thermo Scientific). Frozen DNA samples were placed in microtitre plates and the appropriate primer added before freezing and shipping to Sequetech (Mountain View, CA, United States). Sequence data has been deposited at NCBI (accession numbers are given in Supplementary Table S2).

### DNA Sequence Analysis

Forward and Reverse sequences were aligned using MAFFT 7.0.^1^ Ends were trimmed to generate a common start and end nucleotide for each sequence to be compared. Any ambiguous data were hand checked against the original traces for resolution. All sequences for a particular gene fragment were compared using MAFFT 7.0 and checked for regions of questionable reliability. Those sequences with high background or dubious results (about 2% of the sequences) were discarded or a second sequencing performed. Final sequences were compared using the Neighbor Joining function on MAFFT 7.0 with Bootstrapping set at 500 reiterations. The homologous sequence in *Medicago truncatula* (Medicago genome 4.0 on the Legume Information System at legumeinfo.org) was used as an outgroup for initial analyses, but as these comparisons rarely changed the branching on the resulting cladograms except to greatly compress the branching within the *Pisum* accessions, only unrooted cladograms are presented in the Results section. Trees for 34 genes from Jing et al. (2007) were obtained from the online supplementary material (three other gene trees gave very poor resolution or lacked a *P. s.* ssp. *abyssinicum* sequence and were not used in the current analysis).

### Genetic Mapping

SNP or indel polymorphisms for the 18 genes listed in Supplementary Table S2 and the two histone genes studied by Zaytseva et al. (2012, 2015) were used to locate the genes on the pea linkage map for the JI1794 × Slow RIL population (Weeden et al., 1998). This map now includes over 2000 morphological, isozyme, STS, RAPD and SSR markers, with a resolution of <1 cM in most regions. The location of the genes used by Jing et al. (2007) when not provided by the authors, were approximated by identifying the position of their homologs on the *Medicago truncatula* genomic sequence (Medicago 4.0) and determining the corresponding position on the pea map using flanking markers.

## Results

**FIGURE 1:**
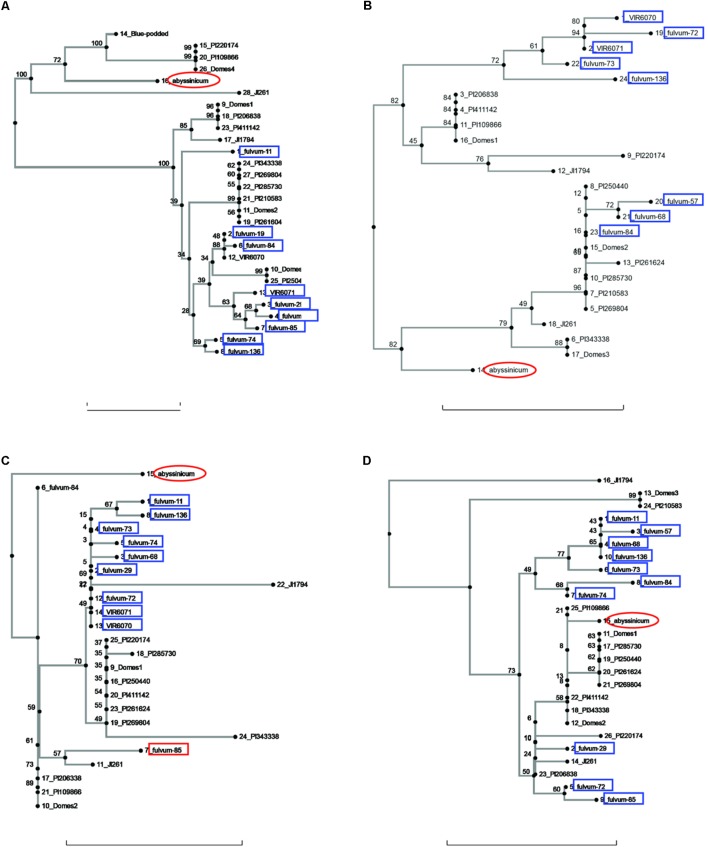
Phylogeny trees of the alleles for **(A)** *Pur*, **(B)** *R*_b_, **(C)** *GlyOH*, and **(D)** *Gpic*. The position of the allele from *P. sativum* ssp. *abyssinicum* is circled in red, those for *P. fulvum* accessions are boxed in blue. The position of each PI accession is presented individually, but those for the modern varieties (except Blue-podded Shelling in **(A)** are lumped into groups labeled Domes1, Domes2, etc). Bar at base of each cladogram represents a nucleotide substitution rate of 0.01.

**FIGURE 2:**
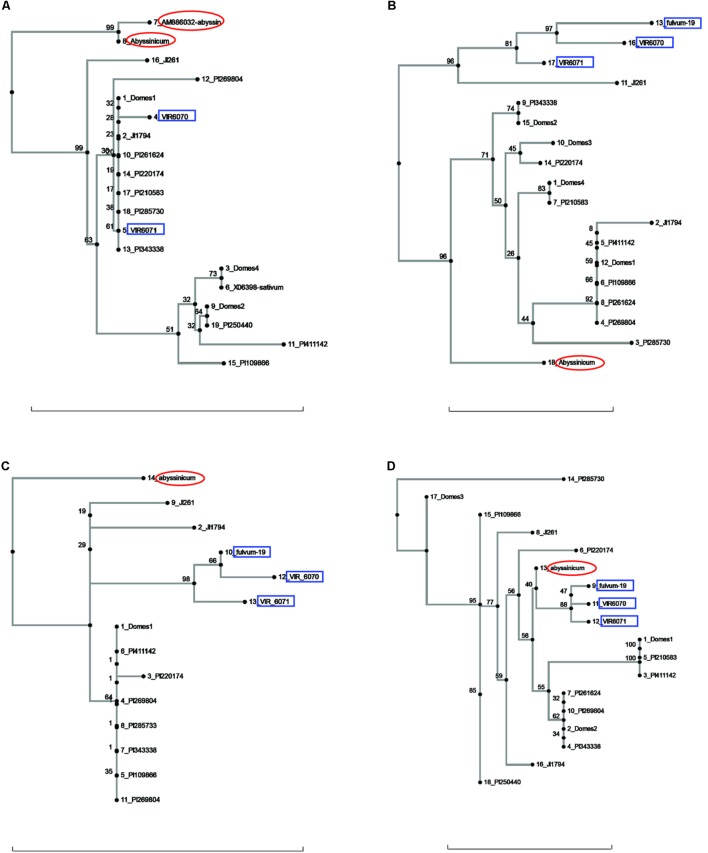
Phylogeny trees of the alleles for **(A)** *Cvc*, **(B)** *Gib2ox*, **(C)** *Er1*, and **(D)** *Skdh*. The position of the allele from *P. sativum* ssp. *abyssinicum* is circled in red, those for *P. fulvum* accessions are boxed in blue. The position of each PI accession is presented individually, but those for the modern varieties are lumped into groups labeled Domes1, Domes2, etc. Bar at base of each cladogram represents a nucleotide substitution rate of 0.01.

**FIGURE 3:**
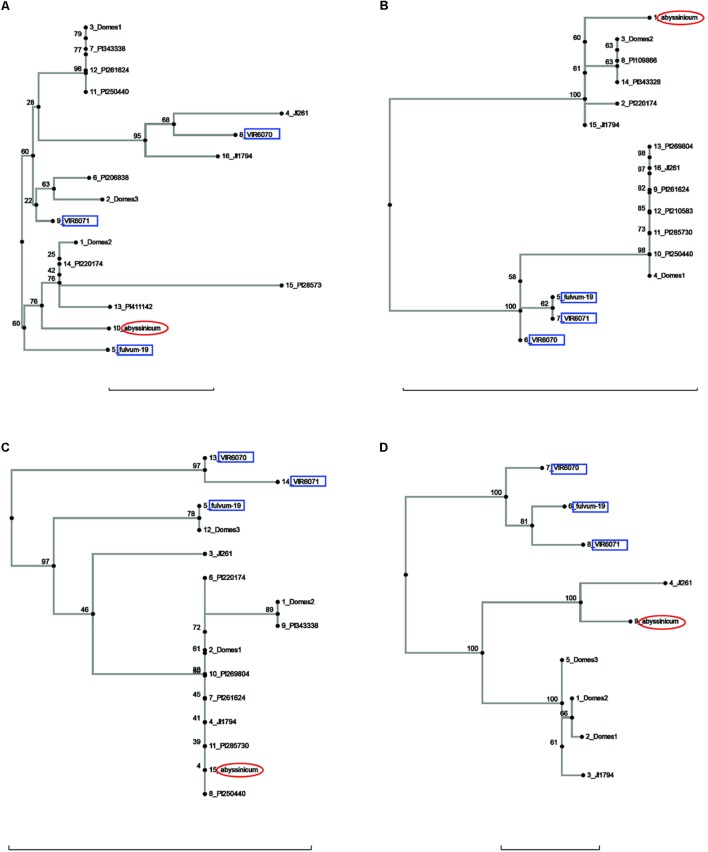
Phylogeny trees of the alleles for **(A)** *Peam5*, **(B)** *Ntfy*, **(C)** *Alat*, and **(D)** *Sbm1*. The position of the allele from *P. sativum* ssp. *abyssinicum* is circled in red, those for *P. fulvum* accessions are boxed in blue. The position of each PI accession is presented individually, but those for the modern varieties are lumped into groups labeled Domes1, Domes2, etc. Bar at base of each cladogram represents a nucleotide substitution rate of 0.01.

Allele trees for each of the genes studied generated by the neighbor-joining process are presented in **Figures 1**–**3** and **Supplementary Figure S1**. In order to make the trees as clear as possible, the bulk of the cultivated accessions have been combined into groups (after the neighbor-joining analysis) and labeled Domes1, Domes2, etc., in order of the size of the group. The alleles within each group are identical or nearly so, and there are no significant branches within a group. The Plant Introduction (PI) accessions representing the four major divisions of genetic diversity in *P. s.* ssp. *sativum* (Kwon et al., 2012) are included as separate entities on all trees, as are the *P. s.* ssp. *elatius* accessions JI 1794 and JI 261, a *P. s.* ssp. *abyssinicum* accession, and three or more *P. fulvum* samples.

Sequences for the bulk of the *P. fulvum* samples were obtained only for four genes (*Pur, Rb, GlyOH*, and *Gpic*). Trees for each of these genes are shown in **Figure 1**. In each tree most of the *P. fulvum* sequences clustered in one region, but in each case there were 2 or 3 sequences that fell outside the cluster. The diverging *P. fulvum* accessions were different for each gene, although fulvum-84 and -85 were divergent in two cases. For *Pur*, *Rb*, and *Gpic*, the sequence for *P. s.* ssp. *abyssinicum* was located in a different part of the tree from any of the *P. fulvum* sequences, indicating that *P. fulvum* was not the source of the allele in the Abyssinian pea. For *GlyOH*, the *P. s.* ssp. *abyssinicum* allele was highly divergent, but branched from the tree between fulvum-84 and a mix of other accessions (**Figure 1C**). In this case the source of the *P. s.* ssp. *abyssinicum* allele is ambiguous, with no close affinities among the germplasm tested.

Several other genes gave ambiguous affinities for the *P. s.* ssp. *abyssinicum* allele (**Figure 2**). In the cases of *Cvc*, *Gib2ox*, and *Er1*, the *P. s.* ssp. *abyssinicum* allele displayed considerable divergence from all other alleles identified. For convicilin, several additional sequences, including one from *P. s.* ssp. *abyssinicum*, were available in the literature. As expected, the *P. s.* ssp. *abyssinicum* sequence from NCBI (AM8886032) was very similar to that obtained in the current study, both possessing relatively large unique insertions as well as several SNPs. A *P. s.* ssp. *sativum* sequence (X06398) was identical to that found in the Domes4 group (**Figure 2A**). However, several other sequences in the literature, one for *P. fulvum* (AM886036), two for *P. s.* ssp. *elatius* (AM66034 and AM66035) and one for *P. s.* ssp. *sativum* (AM66033) all contained a large unique insertion and formed a different branch on the tree with 100% bootstrap support. These sequences were not included on the final tree because they are most likely from an ortholog. The final gene, *Skdh* (**Figure 2D**), branched off the tree between the *P. fulvum* cluster and several *P. s.* ssp. *sativum* groups. However, the tree is compressed in this region, with only 40% bootstrap support for the placement of the *P. s.* ssp. *abyssinicum* sequence. Thus, assessment of phylogenetic relationships is difficult. The *P. s.* ssp. *abyssinicum Peam5* allele also was placed between a *P. fulvum* allele and a *P. s.* ssp. *sativum* cluster (**Figure 3A**), but in this case the three *P. fulvum* accessions were dispersed on the tree, and it would be difficult to assign much significance to the similarity between the *P. s.* ssp. *abyssinicum* and *P. fulvum* alleles.

The sequences of three genes exhibited a similarity between the *P. s.* ssp. *abyssinicum* allele and those found in *P. s.* ssp. *elatius*. If the tree for *Ntfy* is oriented so that the *P. fulvum* cluster is basal, the branches lead to two clusters of accessions, both of which contain *P. s.* ssp. *elatius* and *P. s.* ssp. *sativum* taxa (**Figure 3B**). However, the branch to which the *P. s.* ssp. *abyssinicum* sequence belongs contains the ‘northern humile’ *P. s.* ssp. *elatius* (JI 1794), the sequence from a landrace from Afghanistan (PI 220174) and several other primitive landraces, suggesting that it might represent a group more closely aligned to the original ancestor of the cultivated pea. The tree for Alat (**Figure 3C**) shows a similar pattern, although in this case the *P. s.* ssp. *abyssinicum* allele groups with nearly all the *P. s.* ssp. *sativum*, as well as with JI 1794 and PI 220174. On the other hand, the *Sbm1* allele from *P. s.* ssp. *abyssinicum* clearly groups with the sequence from the ‘wilder’ *P. s.* ssp. *elatius* accession, the northern humile accession grouping with *P. s.* ssp. *sativum* (**Figure 3D**).

The analyses on the remaining six genes all indicate a strong relationship between the *P. s.* ssp. *abyssinicum* allele and alleles in *P. s.* ssp. *sativum* (**Supplementary Figure S1**). The trees all have the same general pattern, with the *P. fulvum* sequences forming a cluster, the *P. s.* ssp. *elatius* sequences usually intermediate between *P. fulvum* and *P. s.* ssp. *sativum*, the *P. s.* ssp. *sativum* in one or more groups, occasionally with one or two associated with a *P. s.* ssp. *elatius* allele, and the *P. s.* ssp. *abyssinicum* allele included within or derived from one of the *P. s.* ssp. *sativum* clusters. In some cases, e.g., *Pgmc* and *Pao*, the *P. s.* ssp. *abyssinicum* allele is highly divergent from the *P. s.* ssp. *sativum* cluster, but it is also distant from any *P. s.* ssp. *elatius* or *P. fulvum* allele.

In order to try to summarize the affinities of the *P. s.* ssp. *abyssinicum* alleles for the various genes, the most closely related sequence(s) to the *P. s.* ssp. *abyssinicum* allele is given for each gene in **Table 1**. Included in this table are the results of 36 comparisons performed previously by others (Jing et al., 2007; Zaytseva et al., 2012, 2015). Of the 54 *P. s.* ssp. *abyssinicum* sequences compared, 12 (11) were closest to a *P. s.* ssp. *sativum* sequence, 6 (5) were scored as basal to *P. s.* ssp. *sativum* (only *P. s.* ssp. *sativum* on branches above), 8 (6) as between *P. s.* ssp. *sativum* and *P. s.* ssp. *elatius*, 20 (16) closest to *P. s.* ssp. *elatius*, 5(3) between *P. s.* ssp. *elatius* and *P. fulvum*, 2 (2) between *P. s.* ssp. *sativum* and *P. fulvum*, and 1 (1) *P. fulvum* (but highly divergent). Numbers in parentheses indicate the total if all those with ‘poor resolution’ marked in the comments column of **Table 1** are eliminated from consideration, thereby avoiding most of the ambiguous data. Either set of results indicate that very little of the nuclear genome of *P. s.* ssp. *abyssinicum* is derived from *P. fulvum*.

**Table 1:** *Pisum* taxon (taxa) with the allele displaying the greatest similarity to the allele in the Abyssinian pea.

Gene	LG	Data source	Subspecies (accession)	Comments
Heme oxygenase	I	Jing et al., 2007	elatius/fulvum	
Pur	I	Current paper	elatius (261)/sativum	
Alatc	I	Current paper	sativum	
EST-202	II	Jing et al., 2007	elatius (3149, 3156)	Fair resolution
EST-176	II	Jing et al., 2007	basal to sativum	
His2-5	II	Zaytseva et al. (2012)	elatius	
PisGen8	II	Jing et al., 2007	elatius (3149)	
His7	II	Zaytseva et al. (2015)	sativum	
EST-189	II	Jing et al., 2007	elatius (1094)	Poor resolution
CvcA	II	Current paper	basal to sativum but divergent	
ACS1	III	Jing et al., 2007	elatius (3147)	
EST-201	III	Jing et al., 2007	elatius (261, 3149)/fulvum	poor resolution
EST-197	III	Jing et al., 2007	sativum/elatius	
Rb	III	current paper	sativum	
Rms1	III	current paper	sativum	
EST-161	III	Jing et al., 2007	elatius (261, 3147)	
Uni	III	Jing et al., 2007	elatius (3156)	
EST-172	III	Jing et al., 2007	elatius/sativum	Poor resolution
EST-198	III	Jing et al., 2007	elatius (1094)	
EST-175	III	Jing et al., 2007	elatius/sativum	poor resolution
Ntfy	III	Current paper	elatius/sativum	
PisGen-7	III	Jing et al., 2007	elatius	
Le	III	Jing et al., 2007	elatius/sativum	
Ko1	III	Current paper	elatius/sativum	
GlyOH	IV	Current paper	elatius/fulvum/sativum	Poor resolution
EST-203	IV	Jing et al., 2007	sativum	Poor resolution
PEAM5	IV	Current paper	sativum/fulvum	
Pim	IV	Jing et al., 2007	elatius (3147)/fulvum (1796)	
EST-163	IV	Jing et al., 2007	sativum	
FRO	IV	Current paper	sativum	
EST-191	IV	Jing et al., 2007	elatius (3149)	
EST-185	IV	Jing et al., 2007	elatius/sativum	Poor resolution
PisGen-5	IV	Jing et al., 2007	elatius (261)/fulvum	
CD859399	V	Jing et al., 2007	elatius (3149, 3155)	
EST-178	V	Jing et al., 2007	basal to sativum	
FBPase	V	Jing et al., 2007	elatius (3149)	Poor resolution
EST-199	V	Jing et al., 2007	elatius	
PisGen 28	V	Jing et al., 2007	sativum	
PisGen-27	V	Jing et al., 2007	fulvum/divergent	
SulTr	V	Current paper	sativum	
EST-196	V	Jing et al., 2007	basal to sativum	Poor resolution
Gib-2-ox	VI	Current paper	basal to sativum	
Er1	VI	Current paper	basal to sativum	
EST-188	VI	Jing et al., 2007	elatius (1094, 3147)	
Gpic	VI	Current paper	sativum	
Sbm1	VI	Current paper	elatius (261)	
PisGen-14	VI	Jing et al., 2007	elatius (261, 3147)	
Pgmc	VII	Current paper	sativum	
SKDH	VII	Current paper	sativum/fulvum	
EST-200	VII	Jing et al., 2007	elatius (1094, 3149)	
EST-171	VII	Jing et al., 2007	elatius	
PisGen 10	VII	Jing et al., 2007	elatius (3149)/fulvum	Poor resolution
PAO	VII	Current paper	sativum	
Starch syn	VI/VII	Jing et al., 2007	elatius (3147)	


The genes in **Table 1** are listed in the approximate order that they have on the linkage map for *P. sati*vum (there are translocations known in *Pisum*, but most of the domesticated germplasm possesses the same karyotype). By examining the changes in the *P. s.* ssp. *abyssinicum* allele affinities as one moves down the column, we can determine if there are any regions of the *P. s.* ssp. *abyssinicum* genome that are primarily derived from *P. fulvum* or from one of the *P. sativum* subspecies. Such an exercise reveals a very low association with *P. fulvum* alleles but indicates approximately even distribution of *P. s.* ssp. *sativum* and *P. s.* ssp. *elatius* affinities. For LG I, the genes investigated cover only the upper half of the linkage group. No clear pattern is evident, all markers being somewhat ambiguous. LG II is reasonably saturated with markers, and most of the alleles indicate a *P. s.* ssp. *elatius* pedigree. The one exception, *His7*, is near the center of the linkage group, a location not known for any genes critical for domestication. The coverage of LG III is very good, and most of the alleles favor a *P. s.* ssp. *elatius* heritage or are ambiguous between *P. s.* ssp. *elatius* and *P. s.* ssp. *sativum*, although a region on the upper portion (around *R*_b_) has a *P. s.* ssp. *sativum* preference. The *P. s.* ssp. *abyssinicum* alleles from genes on LG IV appear to favor *P. s.* ssp. *sativum* sequences except on its lower end. Affinites on LG V and LG VII are about equal, and those on LG VI slightly favor *P. s.* ssp. *elatius*. Such results are consistent with a *P. s.* ssp. *sativum* × *P. s.* ssp. *elatius* hybrid origin of the Abyssinian pea. The *P. s.* ssp. *elatius* accessions that often appear in **Table 1** as having the most similar allele to *P. s.* ssp. *abyssinicum* are from Turkey. The region from Turkey north into Georgia was identified as potentially important in the domestication of pea (Zaytseva et al., 2017). However, much of the *P. s.* ssp. *elatius* germplasm tested by Jing et al. (2007) was originally collected in this country, and the significance of the Turkish connection to the development of the Abyssinian pea is questionable.

## Discussion

### General Comments Regarding the Study

Sequence analysis of 18 gene fragments confirmed the finding of previous studies that there is considerable genetic diversity present in *Pisum* and that the alleles are distributed across taxa, presumably by outcrossing and hybridization. The sequences from the accessions of *P. fulvum* examined here generally formed a cluster, occasionally with one or more accessions outside this cluster and sometimes lying within a *P. s.* ssp. *sativum* or *P. s.* ssp. *elatius* cluster. It is evident that a sample of 11 *P. fulvum* accessions is inadequate to reveal all the sequence diversity within that species for the gene segments used in this study, and a sample size of three certainly limits the conclusions that can be drawn, particularly if the sequences do not form a cluster on the tree such as was the case for *Peam5* (**Figure 3A**). The three accessions of *P. fulvum* chosen as a minimum sample of the species represent those found in preliminary studies to be the most genetically divergent. The conclusion that *P. fulvum* did not make a significant contribution to the *P. s.* ssp. *abyssinicum* nuclear genome is based on the observation that the allele found in *P. s.* ssp. *abyssinicum* never clusters with the common allele of *P. fulvum* whether one considers any of the 18 genes examined in this study or any of the 36 genes that had been subjected to similar analyses previously.

A similar criticism of small sample size could be raised regarding having only two representatives of *P. s.* ssp. *elatius* sequences in the current study. Indeed, no conclusion regarding the relationship between *P. s.* ssp. *abyssinicum* and *P. s.* ssp. *elatius* could have been made had it not been for the availability of the excellent data set of Jing et al. (2007). They included a wide selection of *P. s.* ssp. *elatius* accessions in their study, and it is their data that reveal the close relationship between *P. s.* ssp. *abyssinicum* alleles and *P. s.* ssp. *elatius* alleles in over half the fragments studied. The two studies by Zaytseva et al. (2012, 2015) also included numerous *P. fulvum* and *P. s.* ssp. *elatius* sequences, providing a strong basis for concluding that the *P. s.* ssp. *abyssinicum His2-5* allele is close to those of *P. s.* ssp. *elatius* and the *His7* allele is close to those of *P. s.* ssp. *sativum*.

It may be useful to compare the positions of the *P. s.* ssp. *abyssinicum* allele in each cladogram relative to those for PI 220174. This latter accession represents the subgroup a-2 of Kwon et al. (2012) and more significantly is a sample of a large group of landraces grown in the region between Iran and Nepal. This group is clearly a domesticated pea but appears to represent one of the earliest major branches of *Pisum* germplasm post domestication (Jing et al., 2007). In most of the cladograms, the allele of PI 220174 is identical to an allele found in one of the modern cultivars. In a number of these cases (e.g., **Figures 1A**, **3A–C**) the *P. s.* ssp. *abyssinicum* allele is also identical or very similar. In most other cladograms the allele in *P. s.* ssp. *abyssinicum* is more distant from the alleles in the modern cultivars than is that of PI 220174, as expected if *P. s.* ssp. *abyssinicum* diverged from the modern domesticated germplasm earlier (or was derived from a hybridization with a lineage that had diverged earlier than group a-2). However, in one case, *Gpic* (**Figure 1D**), the allele in PI 220174 is placed between the Abyssinian pea allele and those in *P. fulvum*, contradicting the general pattern. For both the position of PI 220174 and the phylogenetic relationship of the Abyssinian pea with other members of the genus, the general trend appears to be more reliable than results for any particular gene.

The current study contained a large number of accessions of known cultivars, and it is of interest to examine if these cultivars added much to the various gene trees or whether the selected PI accessions would have covered the genetic diversity. A total of six cases can be seen in diagrams (excluding those for *Sbm1* and *FRO1*, for which a complete data set for the PI accessions was not available) where one or more of the pea accessions contained an allele not found in the selected PIs. The unique *Pur* allele found in Blue Podded Shelling (**Figure 1B**) is understandable, because none of the PIs had purple pods. In Alat (**Figure 3C**), Domes4 clustered with a *P. fulvum* accession, and the nearest PI was several nodes away. This Domes4 group consisted of one winter pea variety, ‘Walechia,’ and it must have had some *P. fulvum* in its pedigree. The other four cases (*Pao, Peam5, Cvc*, and *Skdh*) are less easily explained and indicate how difficult it is to capture all the genetic diversity present in modern cultivars in a small set of PIs. Alternatively, there are many instances on the phylogeny trees where at least one PI accession was present, but none of the modern cultivars possessed a similar allele, reflecting the more limited genetic diversity of modern pea varieties.

### Associations With Genes Involved in Domestication

There are two very important changes required for the domestication of a pulse crop: elimination of seed dormancy and elimination of the seed dispersal mechanism (the dehiscent pod). Genetic studies on the dehiscent pod character identified a locus, *Dpo*, on LG III that has a major influence on pod dehiscence (Blixt, 1972). A recessive allele at this locus is primarily responsible for the indehiscent character of most cultivated pea varieties. If the indehiscent pod trait in the Abyssinian pea was derived from an early *P. s.* ssp. *sativum* lineage, one would expect to observe *P. s.* ssp. *sativum* alleles in this region of LG III. In **Table 1**, *Dpo* should be positioned between *Ntfy* and PisGen 7. The *P. s.* ssp. *abyssinicum* allele for PisGen 7 appears to have come from *P. s.* ssp. *elatius*, while the ancestry of the *Ntfy* allele is ambiguous. Should this region be confirmed to be derived from *P. s.* ssp. *elatius* stock, it would imply that *Dpo* mutated independently in the lineage leading to the Abyssinian pea after the divergence of this lineage from that leading to *P. s.* ssp. *sativum*.

The genetic basis of seed dormancy has also been studied in *Pisum*, but with less success. The primary mechanism for dormancy is known to be a thick, impervious testa, however, few studies have reported specific genes influencing this trait, and those genes identified do not appear to have been involved in the domestication process. Unpublished results from a study of the JI 1794 × Slow RIL in my laboratory have suggested that regions near the upper end of LG III and lower end of LG IV may contain genes influencing this character (Brauner et al., unpublished). As the hard-seeded trait is present in JI 1794 and absent in Slow, these could be the genes modified during the domestication of pea. Examination of these regions in *P. s.* ssp. *abyssinicum* (**Table 1**) reveals the *P. s.* ssp. *sativum* allele is present in both the *R*_b_, *Rms1* region of LG III and the EST-163, *FRO1* region on LG IV. The *R_b_/Rms1* region precisely corresponds with the QTL for seed dormancy on LG III, whereas that on LG IV would have centered on the *FRO1*/EST-191 segment. However, should either or both QTL represent a gene from JI 1794 producing a thick testa, the presence of *P. s.* ssp. sativum-related alleles at these locations in the Abyssinian pea would be consistent with the thinning of the testa occurring before the *P. s.* ssp. *sativum* and *P. s.* ssp. *abyssinicum* lineages split.

### Does the Abyssinian Pea Represent an Independent Domestication of *Pisum sativum*?

To address this question, one must first define what is meant by “to domesticate.” Some have defined it as the acquisition of a considerable number of traits that we observed in modern day cultivars (Hammer, 1984; Harlan, 1992), while others see it as the modification of one or two characters that allow man to harvest sufficiently more seed than what was initially planted, thereby allowing a society to feed itself while still maintaining a seed stock for planting the next season (Abbo et al., 2012). Another way of defining the process is the loss of natural adaptations of an organism until it becomes dependent on man for survival. For many crops (e.g., maize, dates, cereal grains, and pulses) domestication has involved changes in reproductive structures and seed dispersal mechanisms (Hammer, 1984). Pulse crops become dependent on man for survival when they can no longer disperse their seeds (possess an indehiscent pod) and cannot maintain a seed bank in the soil to allow survival through years of drought or other factors that prevent seed production during 1 or 2 years (loss of seed dormancy).

Using this last definition, the Abyssinian pea would represent an independent domestication only if seed dormancy and pod dehiscence were independently lost in the two lineages, one leading to *P. s.* ssp *sativum* and one leading to *P. s.* ssp. *abyssinicum*. Convincing evidence pertaining this question remains lacking, but the knowledge that there are several ways to obtain indehiscent pods in *Pisum* (the *dpo* mutation, loss of schlerenchyma in pod wall, making the pod wall thicker with callus growths such as seen in some neoplasm {*Np*} lines), and several avenues to loss of seed dormancy (causing the testa to crack by wrinkling the testa, having the seeds stick to each other, thinning the testa by interfering with phenylpropanoid synthesis) it appears the onus is on those claiming an independent domestication for the Abyssinian pea to demonstrate that the *Dpo* gene and those genes controlling seed dormancy actually did mutate independently in the *P. s.* ssp. *abyssinicum* lineage. The results of the present study provide some evidence for an independent mutation at *Dpo*, but indicate that the lack of seed dormancy may have preceded the divergence of the two subspecies.

It is of interest to ask why *P. s.* ssp. *sativum* has become such a widespread crop while *P. s.* ssp. *abyssinicum* has a very restricted range. The answer may lie in the considerable difference in genetic diversity present in the two taxa. The former has a genetic diversity that rivals that present in maize, whereas the Abyssinian pea is nearly monomorphic at all loci that have been tested. The much greater genetic diversity present in *P. s*. spp. *sativum* undoubtedly facilitates adaptation of the crop to different environments and modification of the actual crop (not just the dry seeds, but also leaves, tendrils, pods, and flowers are known to be eaten). The difficulty in crossing the two subspecies probably prevented breeder/growers in Ethiopia and Yemen from moving traits available in the more widespread domesticate into the Abyssinian pea. Hence, it is now much more appropriate to consider this latter domesticate as a potential, albeit limited source of novel traits for pea improvement.

### The Problem of Highly Unique Alleles Versus Narrow Genetic Diversity in *P. s.* ssp. *abyssinicum*

The Abyssinian pea presents an interesting dilemma when trying to explain its genetic characteristics. Many of its alleles are highly diverged from any allele yet identified in the other *Pisum* taxa, suggesting a very ancient divergence from these other groups, yet the extremely low genetic diversity of *P. s.* ssp. *abyssinicum*, particularly relative to the other *Pisum* taxa, suggests a very recent origin or genetic bottleneck. The unique alleles present in Abyssinian pea are strong evidence against a recent origin (<5000 years bp), whether hybrid or not, because we should still find those alleles in other accessions at the numerous germplasm collections throughout the world. We are left with the genetic bottleneck explanation, with isolation and strong selection being the obvious factors. However, if *P. s.* ssp. *abyssinicum* was widespread at one point, what was its distribution and why, based on how alleles seem to be freely exchanged in the rest of the genus, don’t we see some of the *P. s.* ssp. *abyssinicum* alleles appearing rarely in other accessions?

### Taxonomic Considerations

The taxonomic treatment of a related group of organisms can change as new information is obtained and ideas of what constitutes a particular taxonomic level are modified. Whether only two (Smartt, 1984) or up to six species (Govorov, 1937) should be recognized within the genus *Pisum* has been a moot point since the defining of the genus. Most of the problem exists in trying to further divide the ‘wild’ material referred to as *P. s.* ssp. elatius (*sensu latu*) into species. This germplasm is a diverse assemblage of types, yet the variation appears continuous within this complex (Vershinin et al., 2003; Jing et al., 2007; Kosterin and Bogdanova, 2008; Smykal et al., 2012; Zaytseva et al., 2012, 2015, 2017) and certainly intergrades with *P. s*. ssp. *sativum*. The analysis presented in this paper is consistent with treating ‘sativum’ and ‘elatius’ as a panmictic complex and demonstrates that the *P. s.* ssp. *abyssinicum* germplasm was derived from this complex. If we are not willing or able to define a *P. elatius* at the species level, then what justification is there to define a *P. abyssinicum*?

## Conclusion

The comparison of alleles at 54 genes distributed throughout the pea nuclear genome demonstrated that *Pisum sativum* ssp. *abyssinicum* alleles displayed a much closer relationship to alleles in either *P. s.* ssp. *sativum* or *P. s*. ssp. *elatius* than to those in *P. fulvum*. The possibility that the Abyssinian pea was derived from a *P. sativum* × *P. fulvum* hybridization within the last 10,000 years is thus rejected. The possibility remains that the Abyssinian pea is a product of a *P. s.* ssp. *sativum* × *P. s.* ssp. *elatius* hybridization, with the most likely source of *P. s.* ssp. *elatius* germplasm being Asia Minor. The question whether the Abyssinian pea represents an independent domestication event remains debatable. The indehiscent pod trait required for domestication may have arisen independently in this lineage based on the *P. s.* ssp. *elatius*-related alleles in the *Dpo* region of the *P. s.* ssp. *abyssinicum* genome. However, little evidence is available to determine if the lack of seed dormancy in the Abyssinian pea evolved after this lineage separated from that leading to *P. s*. ssp. *sativum*.

## Author Contributions

The author performed the research and wrote the manuscript.

## Conflict of Interest Statement

The author declares that the research was conducted in the absence of any commercial or financial relationships that could be construed as a potential conflict of interest.

## Footnotes

1 https://mafft.cbrc.jp/alignment/server/

## Supplementary Material

FIGURE S1 Phylogeny trees for the six genes that indicate a close relationship between the allele from the Abyssinian pea (circled in red) and alleles found in *P. sativum* ssp. *sativum*. Alleles from *P. fulvum* accessions are boxed in blue. The position of each PI accession is presented individually, but those for the modern varieties are lumped into groups labeled Domes1, Domes2, etc. Bar at base of each cladogram represents a nucleotide substitution rate of 0.01.
