# Supplementary figures and images for: Domestication of Pea (Pisum sativum L.): The Case of the Abyssinian Pea
Source: Front Plant Sci. 2018 Apr 18;9:515. doi: 10.3389/fpls.2018.00515 (PMC5915832; doi:10.3389/fpls.2018.00515)

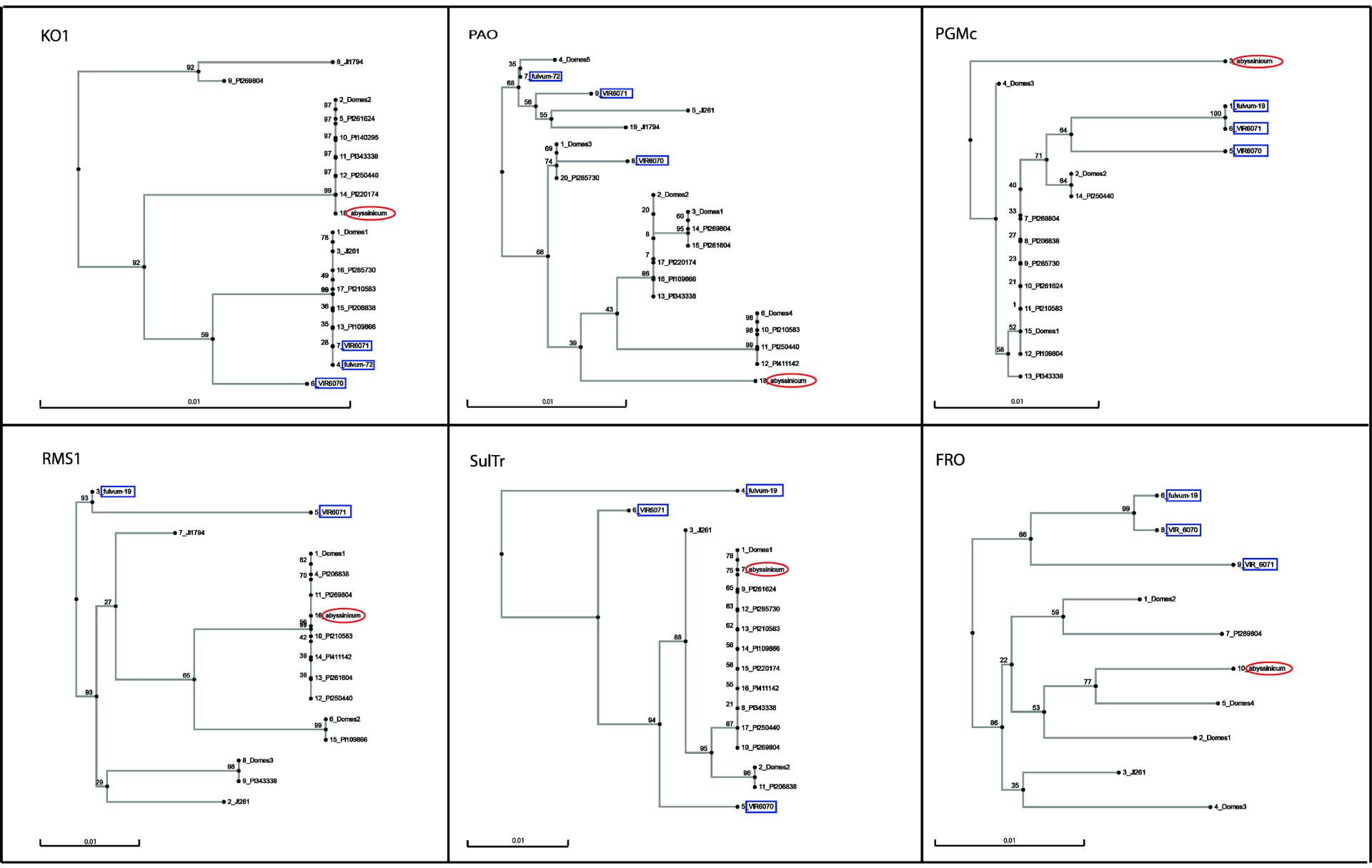

Supplement: FIGURE S1 — Phylogeny trees for the six genes that indicate a close relationship between the allele from the Abyssinian pea (circled in red) and alleles found in P. sativum ssp. sativum. Alleles from P. fulvum accessions are boxed in blue. The position of each PI accession is presented individually, but those for the modern varieties are lumped into groups labeled Domes1, Domes2, etc. Bar at base of each cladogram represents a nucleotide substitution rate of 0.01. [file Image_1.tif]
