# Supplementary material for: Domestication of Pea (Pisum sativum L.): The Case of the Abyssinian Pea
Source: Front Plant Sci. 2018 Apr 18;9:515. doi: 10.3389/fpls.2018.00515 (PMC5915832; doi:10.3389/fpls.2018.00515)
Supplement: Supplementary file 2 [file Table_1.docx]

Supplementary Table 1. *Pisum* germplasm examined

Cultivated accessions of *Pisum sativum* ssp. *sativum*

Admiral Dakota Mozart

Agassiz Darien Navarro

Alaska Dark Skin Perfection Neptune

Almota Delta Novella II

Alsweet Early Columbia Old Muffin

Amarillo Early Freezer Oregon Sugar Pod

Ambassador Encore Orka

Amigold Frimousse Perfect Freezer

Aragorn Frisson Primo

Arcadia Gain Progress #9

Arvika Green Arrow Ranger

Atlas Golden St. Mauren

Badger Gunner Salamanca

Bohatyr Hyline Scout

Bolero K2 Shamrock

Bonneville K90-2131 Spider

Blue-podded Shelling Lifter Stirling

Bridger Little Marvel Striker

British Wonder Lincoln Sutton’s Harbinger

Carneval Majoret Telephone

Champion of England Mars

Cooper Mexique

Cruiser Miragreen

USDA Plant Introduction accessions and

PI 109866 Venezuela Group b of Kwon et al. (2012)

PI 206838 USA Group d of Kwon et al. (2012)

PI 210583 USA Group b of Kwon et al. (2012)

PI 220174 Afghanistan Group a-2 of Kwon et al. (2012)

PI 250440 Czech Republic Group b of Kwon et al. (2012)

PI 261624 Spain Group c of Kwon et al. (2012)

PI 269804 United Kingdom Group c of Kwon et al. (2012)

PI 285730 Poland Group c of Kwon et al. (2012)

PI 343338 USA Group d of Kwon et al. (2012)

PI 411142 New Zealand Group d of Kwon et al. (2012)

*Pisum elatius* accessions

JI 261 Southern Turkey Group a-1 of Kwon et al. (2012)

JI 1794 Golan Heights, Israel Group a-1 of Kwon et al. (2012)

*Pisum fulvum* accessions

VIR 6070

VIR 6071

P. fulvum-11

P. fulvum-19

P. fulvum-29

P. fulvum-68

P. fulvum-72

P. fulvum-74

P. fulvum-84

P. fulvum-85

P. fulvum-136
